# Modeling allosteric signal propagation using protein structure networks

**DOI:** 10.1186/1471-2105-12-S1-S23

**Published:** 2011-02-15

**Authors:** Keunwan Park, Dongsup Kim

**Affiliations:** 1 Department of Bio and Brain Engineering, KAIST, S. Korea

## Abstract

Allosteric communication in proteins can be induced by the binding of effective ligands, mutations or covalent modifications that regulate a site distant from the perturbed region. To understand allosteric regulation, it is important to identify the remote sites that are affected by the perturbation-induced signals and how these allosteric perturbations are transmitted within the protein structure. In this study, by constructing a protein structure network and modeling signal transmission with a Markov random walk, we developed a method to estimate the signal propagation and the resulting effects. In our model, the global perturbation effects from a particular signal initiation site were estimated by calculating the expected visiting time (EVT), which describes the signal-induced effects caused by signal transmission through all possible routes. We hypothesized that the residues with high EVT values play important roles in allosteric signaling. We applied our model to two protein structures as examples, and verified the validity of our model using various types of experimental data. We also found that the hot spots in protein binding interfaces have significantly high EVT values, which suggests that they play roles in mediating signal communication between protein domains.

## Introduction

Due to the dynamic nature of protein structures, cooperativity and allostery are known to be important regulatory systems of protein activity [1-4]. Allosteric regulation can be induced by the binding of effective ligands, mutations or covalent modifications (so-called allosteric effectors), which perturb sites distant from the signal initiation site. Furthermore, these perturbations often induce important changes in protein structures, such as structural changes in the region around an active site, which increase or decrease ligand binding affinity [1,5-7]. Historically, allosteric regulation has typically been defined in quaternary structures (e.g., hemoglobin), but now it is recognized as an intrinsic property of all proteins, including monomeric structures [8].

Understanding allosteric regulation is of great interest. However, the mechanisms that underlie distal site-to-site communication remain largely elusive. Therefore, it is important to determine how the induced signal is transmitted via an amino acid network and how the allosteric effects are represented in a protein structure. Based on this notion, many researchers have attempted to develop models for describing intramolecular signaling pathways using different sources of information [9-15].

Recently, del Sol et al. reviewed important allosteric modulations via multiple signaling pathways [16]. They suggested that allosteric signals are transmitted through multiple and pre-existing pathways on the structural ensemble. Indeed, many in-depth atomic structural studies, including the NMR relaxation method [17,18], have proposed that allostery is a multifaceted phenomenon caused by cooperative site-to-site communication through multiple paths [6,16,19-25]. Therefore, a simple path connected by a few residues which was often described in previous studies [10,11,26] is unlikely to describe an allosteric mechanism. Instead, combinatorial and cooperative effects involving multiple pathways need to be considered.

In this work, we developed a method to model allosteric communication by considering all possible signaling routes. The presented method used a protein structure network constructed based on the intrinsic assumption that network centrality measures (i.e. relative importance of a node within the network) represent various methods of signaling transmission [27]. In our model, the global perturbation effects from a particular signal initiation site were estimated by calculating the expected visiting time (EVT), which describes the signal-induced effects caused by signal transmission through all possible routes. We hypothesized that the residues with high EVT values play important roles in allosteric signalling, and found experimental evidences supporting the idea.

## Methods

### Binding hot spot data

Experimental binding data (ΔΔG values) of alanine-mutated interface residues of known protein complexes have been mostly widespread for estimating the residue-contribution to the binding event (i.e. finding the binding hot spots). In this study, ΔΔG data collected from Cho’s study [28] were used to investigate the relationship between the EVT and the binding hot spots, and to construct regression model. From the data, the protein entries that had less than three mutations were removed due to the meaningless correlation for two data points (the correlation should be always one), which left 13 protein complexes with 222 mutations.

### Network representation of protein structure

A protein structure can be represented by a network composed of nodes and edges. In this network, nodes are amino acids and are connected by edges when the mutual distance of the inter-residue atom pair is within specific distance cutoffs. The distance cutoffs used in the present study ranged from 3 Å to 12 Å. Ligands are also regarded as nodes when they bind to the protein structure. The edge weight is given by an affinity value, assuming that nodes with higher affinity have higher interaction strengths. In this study, the affinity value between residue *i* and residue *j* (*a_ij_*) was defined as described in Chennubhotla et al. [29] as follows:

,

where *N_i_* and *N_j_* are the numbers of heavy atoms (all but hydrogen) in residues *i* and *j*, respectively, and *N_ij_* is the total number of atom-atom contacts within a distance cutoff between the two residues. The affinity values between the nodes that did not satisfy the contact criterion were set to zero. This representation assumes that the strong (weak) interactions occur between residue pairs with large (small) numbers of atom–atom contacts [29]. The terms in the denominator were used to remove biases due to size effects.

### Calculation of EVT based on a random walk

Conceptually, signals based on a random walk determine the next step in probability. Thus, once the signal-initiating and signal-absorbing sites are determined, the trajectory by which the signal travels can be recorded mathematically as the visiting frequency of the signal. Thus, the EVT value for node ‘X’ can be said to be the average visiting frequency of signals that pass through node ‘X’ for all absorbing sites. These relationships and mathematical formulas are described below.

First, the weighted protein structure network was transformed into Markov transition matrix **T**, which determines the outgoing probability of the signal to the neighboring nodes on the next step. The transition probability from node *i* to node *j* (*T_ij_*) is given by the affinity value between nodes *i* and *j* divided by the sum of the affinity values (degree) for node *i*:

,

where *d_i_* is the degree of node *i* and *a_ij_* is the affinity value between *i* and *j* nodes. Here, the self-edge was not considered.

Next, imagine that a signal initiated from node *i* travels through intermediate nodes until it finally reaches the absorbing node *k*. Because matrix **T** represents the direct transitions to the neighboring nodes (one-step transition), we can consider multiple-step transitions by multiplying matrix **T** multiple times under the constraint that signal transmission stops when it reaches the absorbing node *k*. This information flow can be modeled with the standard absorbing Markov chain model [30], in which the *n*×*n* matrix is particularly important and known as the ‘fundamental matrix’ of the corresponding adsorbing Markov chain, **F**:

,

where **T***^k^* is the reduced transition matrix after the *k*-th row and *k*-th column were removed from matrix **T**. It has been shown that the matrix (**I** - **T***^k^*) is invertible if all nodes are connected [31]. The matrix element  represents the expected number of times that the intermediate node *j* is visited during a random walk from node *i* to node *k*.

Finally, the EVT for each node was calculated by averaging **F***^k^* over all absorbing nodes (*k*) as follows:

,

where *n* is the number of nodes in the protein structure network. For convenience, when calculating **M**, the *k*-th row of **F***^k^* was filled with 0 (not emitting) and the *k*-th column was filled with 1 (entering once). Then, the *i*-th row vector of matrix **M** contains the EVT values for each node when the corresponding *i*-th node is selected as a signal-initiation node. In the end, the row vector of matrix **M**, which we called the EVT profile, was used to mimic the perturbation effects by an initiation signal.

**Figure 1:**
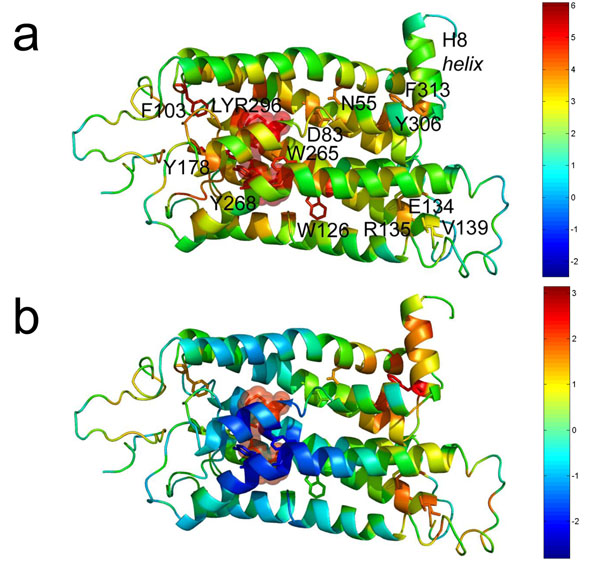
(a) The EVT values and (b) scaled EVT values (by the shortest path length) of a GPCR (rhodopsin) are represented with colors (red: higher EVT, blue: lower EVT). The sphere in each protein structure represents the signal initiation site, and the labeled residues are shown as sticks.

**Figure 2:**
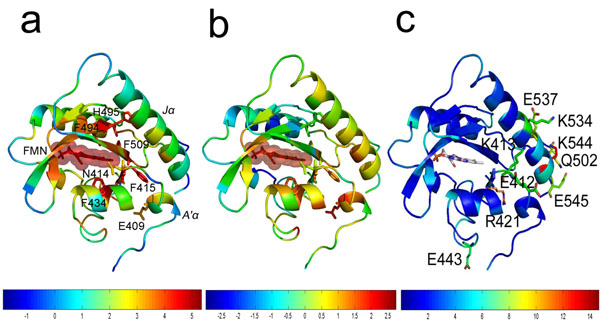
(a) The EVT values and (b) scaled EVT values (by the shortest path length) of the PAS domain are represented with colors (red: higher EVT, blue: lower EVT). The sphere in each protein structure represents the signal initiation site, and the labeled residues are shown as sticks. (c) The comparison of EVT profiles between CLS and CDS is shown with colors. The ratio of the EVT values between CLS and CDS [(CLS/CDS)-1] was used to color the structure to show the different signaling effects of each residue. The red color indicates a larger difference between CLS and CDS.

Furthermore, the evaluated EVT values were normalized for each protein structure network as z-scores (Figure 1 and Figure 2) to indicate whether the EVT values were above or below the mean in unit of the standard deviation as follows:

,

where x is a raw EVT score to be normalized, *µ* is the mean and σ is the standard deviation of the EVT values. Thus, the mean and standard deviation of the EVT values for each protein structure were 0 and 1, respectively.

**Figure 3:**
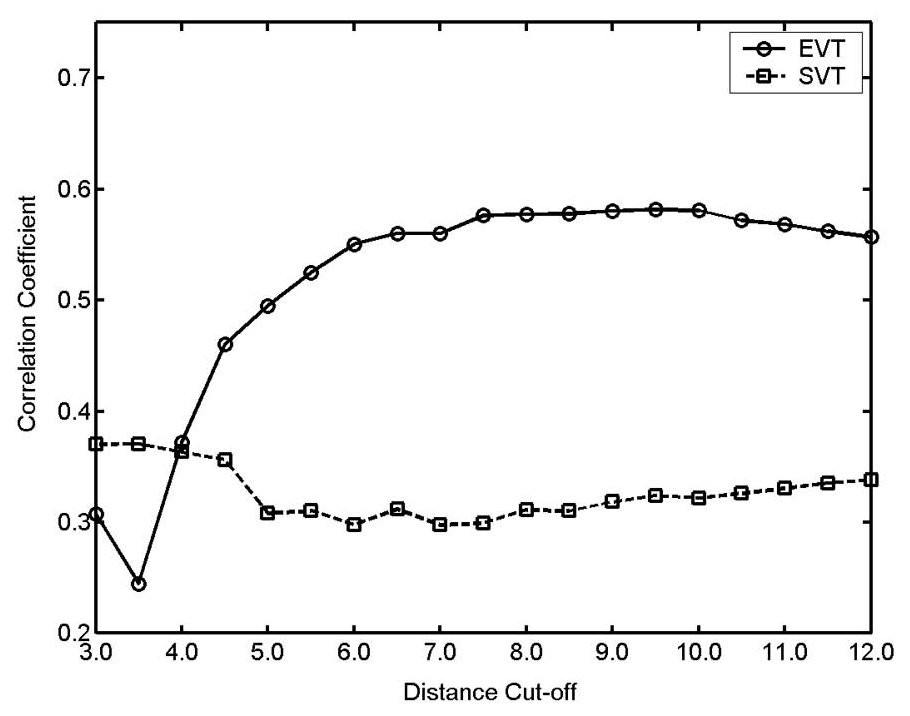
Average correlation coefficients (EVT and ΔΔG values) for each model are shown as the different distance cutoff values. The abbreviations in the figure are EVT (the model based on EVT) and SVT (the model based on SVT). The prediction performance using EVT is much higher than SVT, which suggests that random walk is more realistic model than the shortest path.

The shortest path-based model was also constructed using the same method for the EVT profile except that the signal visiting time for each node was calculated and counted on the shortest paths. Briefly, the shortest paths from a starting node to every absorbing node were recorded, and the number of paths that pass through a particular node was counted. This shortest path visiting time (SVT) for each node was used for comparison with the EVT value (Figure 3).

### Average signal traffic of all pairwise signal transmissions

For finding the average high signal traffic nodes, we calculated the average EVT profile for all starting nodes as a typical calculation of network centrality measures. The average EVT profile is conceptually the same as betweenness centrality[32], which measures how frequently the node is used as an intermediate node during all site-to-site communication events. Thus, a high average EVT value indicates that a node is highly visited and acts like a bridge. The average EVT profile was simply calculated by summing all row-vectors of matrix **M** because one row-vector represents the EVT profile from one signal initiation node to every absorbing node.

### A linear regression model for predicting binding hot spots

A linear regression model was generated to elucidate the relationship between EVT values and binding free energy changes (ΔΔG). The ΔΔG value between the mutants and wild type was measured by replacing the corresponding residues with alanine and measuring the effect on binding [28], which indicates the energetic contributions of individual side chains to protein binding. For this procedure, the *lm* function of the *stat* package in R (http://www.r-project.org) was used, and the quality of the regression model was accessed using the residual standard error (the response minus the fitted value) and p-value. In addition, the correlation coefficient between the fitted values and the real free energy changes was also used.

## Results

### Different networks by different distance cut-offs

Before discussing the signaling effects predicted by our model, it would be worth discussing the effect of different distance cutoff values on the network analysis because different network connectivity often produces significantly different results [33]. This type of ambiguity due to network rewiring often arises in the field of network analysis and makes it difficult to interpret the biological meaning. For example, the relationship between degree centrality and gene essentiality in the yeast protein interaction network has been dramatically changed as new connections have been detected and added [32]. Therefore, it is important to select a robust cutoff value that is insensitive within a certain reasonable range and to select the most meaningful value that shows higher performance in appropriate test cases. After considering these aspects, a cutoff value of 8 Å was used in this study because it had longer coverage (than previous studies [26,33,34] that used cutoffs of around 5 Å), high robustness, and high predictability of binding hot spots (see later sections).

The long-range cutoff is more suitable if the network is connected by weighted edges as in the present study because the transition matrix from a weighted network would not be changed significantly as the cutoff values are changed. In fact, the EVT values of different cutoff values in our examples were highly correlated. For example, the correlation coefficients between 6 Å and 12 Å were higher than 0.8 (Additional file 1). The performances of the predicted binding hot spots also showed similar results to the performances in the range of 6 Å and 12 Å (Figure 3). In addition, by using the long cutoff value, effects due to the long-range interactions caused by dynamic movements could be included in our model. Accordingly, the cutoff value of 8Å was consistently used for all proteins analyzed in this study.

### EVT profiles of two protein structures

Perturbation effects induced by a signal initiation site were estimated by calculating the EVT profiles for two protein structures: G protein-coupled receptor (GPCR) and Per/Arnt/Sim (PAS) domain. The EVT profile contains information on all possible signaling routes by a stochastic process, and the resulting effects are displayed in Figure 1 and Figure 2. In Additional file 2, EVT values for the protein structures are listed. We also gave special attention to the residues that were located far away from the signal initiation sites and simultaneously had high EVT values because they are potential allosteric regulatory sites. To clearly represent the distant but high EVT residues with colors, EVT value of each residue was multiplied by the shortest path length from the signal initiation site. The protein structures with this scaled EVT value are shown in Figure 1b and Figure 2b (also after z-score normalization), where red residues have high EVT values and are located long distances from the signal initiation site.

### G protein-coupled receptor (GPCR)

GPCRs comprise a large protein family of membrane-bound receptors. Because they are often involved in transferring cellular signals and inducing signaling cascades, they have been regarded as primary drug targets. As a result, many studies have attempted to elucidate their structural features and signaling mechanisms [19,35]. Furthermore, because the external signals (e.g., ligand binding or light absorption) that cross the plasma membrane are thought to trigger structural changes at distant regions and activate cellular signaling [36], these long range interactions of the amino acid network have also been studied previously [9].

In this study, we used the rhodopsin receptor as a representative GPCR (PDB id: 2j4y, A chain) [37]. Its structure consists of intradiscal (corresponding to the extracellular domain of related GPCRs), membrane-embedded and cytoplasmic surface domains. The intradiscal domain is mainly composed of loops that connect seven transmembrane helices (H1-H7) of the membrane-embedded domains, and the interhelical networks of the transmembrane helices are known to play a central role in activating rhodopsin. The cytoplasmic domain also consists of many loops and a short α-helix (H8) that lies nearly perpendicular to H7 and is thought to be involved in G-protein binding [23]. Moreover, the retinal binds covalently to Lys296 on the intradiscal portion of the transmembrane helix through a Schiff base linkage, and its light-dependent isomerization induces cellular signaling. Thus, the EVT profile for LYR296 (retinal conjugated Lys296 in H7 helix) was calculated in this case.

The EVT values (represented by different colors in Figure 1a) suggest that the residues near LYR296 tend to have higher signal transmission. For example, the residues with high EVT values were Tyr178, Tyr268, Phe103, Trp126 and Trp265 (2.71, 2.29, 2.25, 2.23, and 2.21, respectively), and most of them participate in hydrophobic packing with LYR296 near the intradiscal domain. The side chains of Tyr178, Tyr268 and Trp265 constrain the position of LYR296, and Trp126 and Trp265 were shown to reorient to more polar environments during receptor activation. Specifically, photoactivation of rhodopsin involves a change in the relative positions of H3 and H6, which contain Trp126 and Trp265, within the α-helical bundle of the receptor [23].

In addition, some residues in an interhelical packing region distant from LYR296 also had high EVT values. Among them, Asp83 (1.35) formed hydrogen bond interactions with Asn55 (1.32), which is known to play a central role in the hydrogen bond network between the helices[23]. Tyr306 (1.39) is in the highly conserved Asn/Pro/X/X/Tyr motif (Asn302/Pro303/Val304/Ile305/Tyr306) [23] that directly interacts with another highly conserved residue, Phe313 (1.32). The two residues form a hydrophobic interaction in the amphipathic H8 helix, which implies that they are functionally and allosterically important (Figure 1b). For example, structural changes were detected at positions Tyr306, Phe313, and Cys316, which is consistent with movements of the nearby helix H6, and a light-dependent interaction was observed with Cys316 [21,38].

Arg135 is located on the opposite side of the protein from helix H8 and had a high EVT value (1.07), even though it is far from LYR296. Arg135 belongs to the highly conserved E/DRY motif in H6 that is involved in the major structure perturbations associated with the transition to the active state [35]. The hydrogen-bonding network that links H3 and H6 involves the conserved residue Arg135, which interacts with Glu134. Glu134 forms a salt bridge with Arg135, and this Glu134/Arg135 di-peptide likely forms a functional domain that is responsible for inducing the release of GDP [23]. In addition, Val139 also had a relatively high EVT value (0.44), even though it is far away from LYR296 (Figure 1b). Interestingly, three consecutive Val residues (Val137, Val138, and Val139) are known to form a cytoplasmic cap on helix H3. The Val tri-peptide might stabilize the Glu134/Arg135 salt bridge, which in turn maintains the receptor in its off state in the dark; this implies that these residues are highly connected to LYR296.

### Per/Arnt/Sim domain (PAS)

Phototropins are flavin-based photoreceptors that regulate phototropism (the ability of plants to bend toward sunlight). They are composed of two N-terminal light, oxygen, or voltage (LOV) domains, denoted LOV1 and LOV2, and a C-terminal serine/threonine kinase domain. LOV2 is known to be the predominant photoreceptor domain that modulates light-dependent autophosphorylation of the kinase domain. In addition, LOV2 is a typical PAS domain and binds to the flavin mononucleotide (FMN) chromophore, which absorbs light and then transmits the signal through the structure. That is, the formation of the covalent bond between Cys450 and FMN and the light-induced rearrangement of the FMN binding pocket have been proposed to modulate its enzyme activity. This signaling event is also known to propagate across the β-sheet to the hydrophobic interface formed by the core LOV2 domain, the N-terminal turn-helix-turn motif (A’ α helix), and the Jα helix, resulting in the conformational changes at the site near the Jα helix [25]. The two helices (A’ α helix and Jα helix) pack against the surface of the β-sheet of the core LOV domain and are stabilized by amphipathic interactions and a conserved hydrogen-bonding network. Therefore, there may be some functional relationship between these helices and the hydrophobic patch on the surface of the β-sheet of the core LOV domain. Harper et al. showed that the structural change caused by light-induced rearrangement of hydrogen bonds near FMN propagates to both the N-terminal motif (A’ α helix) and C-terminal flanking regions (Jα helix)[39]. Thus, in this example, FMN was used as a signal initiation site and the cryo-trapped dark structure (2v0u) was used for constructing the protein structure network. In addition, the EVT profiles between the cryo-trapped dark structure (CDS, PDB ID: 2v0u) and light structure (CLS, PDB ID: 2v0w) were compared to observe the differences induced by light-dependent signaling.

Generally, the hydrophobic residues near FMN and the residues between the Jα helix and central β-sheets had high EVT values (Figure 2a). The result suggests that the internal hydrophobic packing region of the amphipathic Jα helix has an important effect on signal transmission from FMN to the Jα helix, which is consistent with previous studies [25]. Among them, Phe415, Phe494, Phe434, Phe509 and His495 had high EVT values (2.02, 1.95, 1.80, 1.73, and 1.64, respectively). Phe434, Phe494 and Phe509 near the bound FMN form the ligand-binding pocket. They also provide the hydrophobic docking sites for the Jα helix (Ile510 and Val512) or A’ α helix (Phe415).

However, Phe415 and His495 are located in opposite directions compared to the above three residues. Among them, Phe415 directly interacts with the N-terminal A’α helix (Thr407-Arg410). Interestingly, Glu409 (EVT: 0.87), which is far from FMN (Figure 2b), is highly conserved in the PAS domain and stabilizes the N-terminal A’ α helix through the hydrogen-bonding network with neighboring residues (e.g., Asn432 and Arg442), which prevents further displacement of the A’ α helix [39]. Because it is known that the interactions between the LOV2 core domain, A’ α helix and J α helix (which flanks the LOV2 core domain) play an important role in light-mediated signal propagation, the stabilizing interaction of Glu409 appears to be critical for signaling. On the other hand, His495 interacts with the middle portion of the Jα helix and forms a hydrogen-bonding network between the LOV2 domain and helix Jα (with Glu475, Thr495, Gln497 and Lys533). Interestingly, the importance of this middle part of Jα has been demonstrated by previous studies [25,39].

The comparison of EVT profiles between the CLS and CDS shows a more intuitive view (Figure 2c). Although the overall structures of CLS and CDS are almost identical (rmsd: 0.20Å), the dark minus light difference Fourier maps constructed by Halavaty et al. showed significant structural changes in the FMN binding pocket, the middle part of the Jα helix and the N-terminal turn-helix-turn motif (A’α helix) [25]. In addition, they also found that the displacements are caused by light-dependent disruption of the Asn414-Asp515 hydrogen bond (present in CDS) after Cys450-FMN interaction formation. Similar to the dark minus light difference Fourier maps, the ratio of the EVT values between CLS and CDS, , was used to investigate the different signaling effects of each residue (Figure 2c). The result shows that the EVT values of the Jα helix (Gln502, Lys534, Glu537, Lys545 and Glu546) and the loop (Glu412, Lys413, and Arg421) adjacent to the A’α helix were significantly altered. On the contrary, the hydrogen-bonding network of Glu409, Asp432 and Arg442 (not shown) showed little difference in EVT values, which is consistent with their unchanged positions in both CDS and CLS. Accordingly, our results showed overall consistency with the dark minus light difference Fourier maps.

### Binding hot spots of the protein complex and their higher signal traffic

Protein-protein interactions have been analyzed in terms of network properties, such as hubs and clusters of amino acid residues in the protein complex, with particular focus on the protein-protein interface and binding hot spots [40-42]. This approach gives a global perspective of the interactions across the interface, which is difficult to obtain from pairwise interaction or loss of accessible surface area analyses. For example, Morra et al. tried to elucidate the mechanisms of signal propagation and determine the hot spots involved in interdomain communication pathways [43]. In addition, Fenton discussed allosteric interactions, such as binding hot spots, that do not elicit an allosteric effect on the binding of a second ligand [44].

Similarly, the binding hot spots of the protein complex were examined in terms of signal transmission in a protein structure network. We hypothesized that the binding hot spots act as a signal transmitter between interacting protein domains and thus will have higher signal traffic than others. Because ligand binding to one protein domain often induces allosteric changes in the interacting domain, such as in the caspase-1 dimer, a perturbing signal should be efficiently propagated across the binding interface. Furthermore, the interface residues that have higher signal traffic may have much greater effects on protein binding.

To test this hypothesis, experimental binding data representing the binding free energy change (ΔΔG value) were collected, and the relationship between the ΔΔG value and the average EVT value (average EVT across all initiation sites; for details see Methods) was investigated by constructing a linear regression model. The results show that all linear regression models had positive correlations that ranged from 0.31 to 0.89 (Figure 3 and Additional file 3), which suggests that the nodes that have higher effects on protein binding (higher ΔΔG) tended to have high EVT values. In addition, among the 13 linear regression models, 8 models had significant p-values (F-statistic for linear regression model) below 0.1 (Additional file 3). Accordingly, the results not only validate our model but also support our hypothesis that binding hot spots have higher signal traffic between interacting domains.

### Effect of distance cut-off values on predicting binding hot spots

The models based on EVT were affected by the distance cutoff values in short cutoff ranges. That is, the long distance cutoffs from 6Å to 12Å usually showed high robustness (little variation) and high correlation with the ΔΔG values, but cutoffs below 6Å showed lower correlation with dramatic performance changes (Figure 3). Accordingly, the long-range cutoff values would contain more information about dynamic movements and appear to describe the signaling events within a protein structure better than shorter ones.

## Conclusions

We demonstrated that the EVT profiles are largely consistent with experimental data including binding hot spot prediction, which suggested that the residues with high EVT values have important roles in allosteric signaling regardless of their physical distance from a signal initiation site.

However, it also should be noted that a high EVT value (or high signal traffic) of a particular residue is not sufficient to indicate an allosteric change in the residue; i.e., EVT value reveals the importance of the sites for an allosteric change by a particular perturbation rather than predict how the conformation of a perturbed residue is changed.

In addition, our model cannot consider dynamic large-scale changes in protein structure but only a static and single protein structure. Although, to alleviate this limitation, we used a much larger cutoff value (8Å) than has been used in many previous studies, future network models of protein structures must consider the multiple structural variations and resulting different network topologies.

## Competing interests

The authors declare that they have no competing interests.

## Supplementary Material

Additional file 1 EVT correlations between different distance cut-offs among the six proteins including GPCR and PAS domain Click here for file

Additional file 2 Z-transformed EVT values for GPCR and PAS domain are listed Click here for file

Additional file 3 Thirteen linear regression models of the average EVT and ΔΔG values for each protein structure network Click here for file
