# Supplementary figures and images for: Modeling allosteric signal propagation using protein structure networks
Source: BMC Bioinformatics. 2011 Feb 15;12(Suppl 1):S23. doi: 10.1186/1471-2105-12-S1-S23 (PMC3044278; doi:10.1186/1471-2105-12-S1-S23)

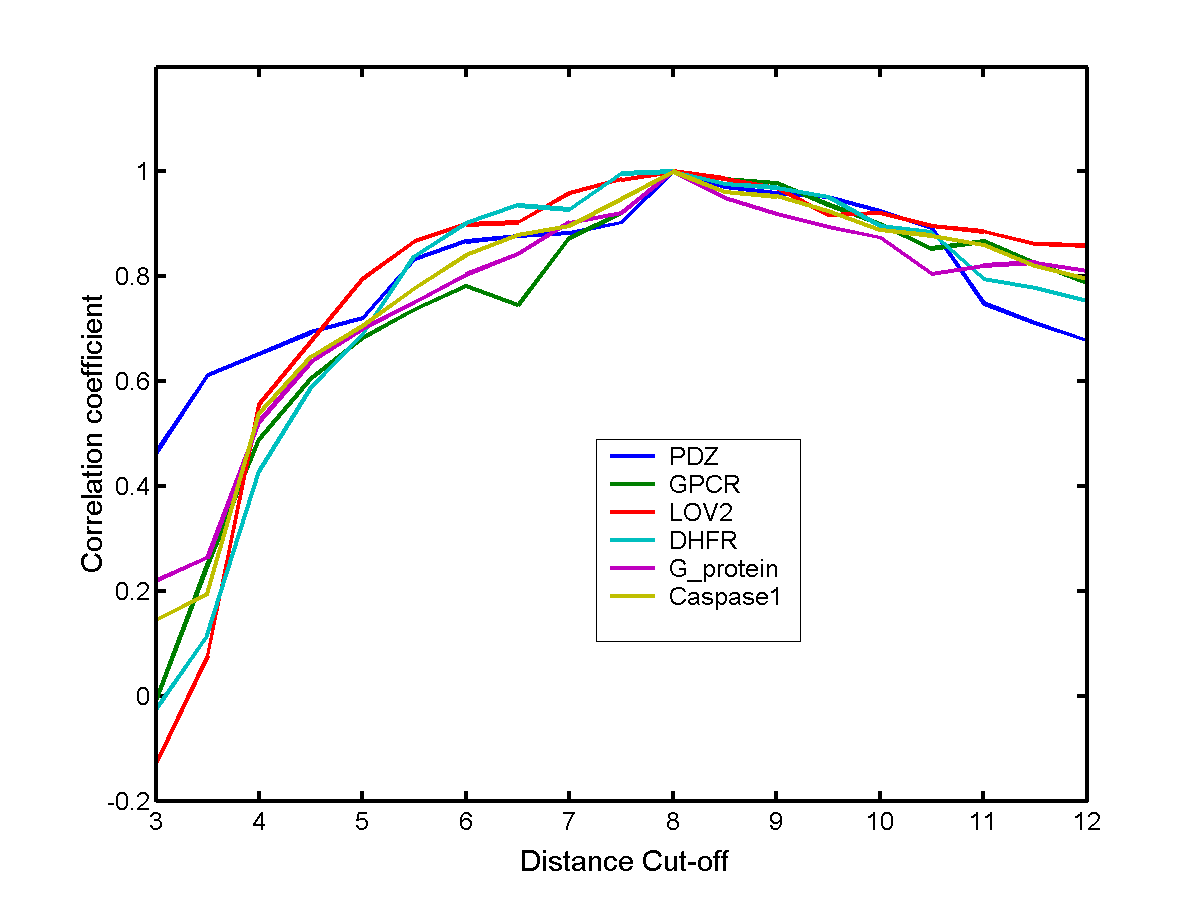

Supplement: Additional file 1 — EVT correlations between different distance cut-offs among the six proteins including GPCR and PAS domain [file 1471-2105-12-S1-S23-S1.tif]
