# Supplementary material for: Modeling allosteric signal propagation using protein structure networks
Source: BMC Bioinformatics. 2011 Feb 15;12(Suppl 1):S23. doi: 10.1186/1471-2105-12-S1-S23 (PMC3044278; doi:10.1186/1471-2105-12-S1-S23)
Supplement: Additional file 2 — Z-transformed EVT values for GPCR and PAS domain are listed [file 1471-2105-12-S1-S23-S2.doc]

Supp2. Normalized EVT values (8.0 A distance cut-off) for the two protein structures (PAS: 2v0u and GPCR: 2j4y)

**[PAS: 2v0u, mean 216.33, sd 78.66]**

A1547FMN 5.34296740774673

A401GLY -1.88896426632801

A402GLU -1.40428998790928

A403PHE -0.716759637536799

A404LEU -1.4085914938154

A405ALA -0.918536785866858

A406THR -0.627609042425923

A407THR -1.27474433254467

A408LEU 0.241245404937882

A409GLU 0.87489630553274

A410ARG -0.608107416689272

A411ILE -0.105972447132397

A412GLU -1.45394529350797

A413LYS -0.0376978971400501

A414ASN 0.717106501657184

A415PHE 2.01771298842471

A416VAL 1.01612672776173

A417ILE 1.21839966329246

A418THR 1.12909220283652

A419ASP 0.37300826394406

A420PRO 0.00621090744138429

A421ARG -1.45420743440575

A422LEU -0.580695395195779

A423PRO -1.18000937867005

A424ASP -0.478570220104718

A425ASN 0.834262417430813

A426PRO 0.857161591290538

A427ILE 1.22906679138384

A428ILE 0.250686162798938

A429PHE 0.581890480543884

A430ALA 0.554938566898203

A431SER 0.828696175989211

A432ASP 0.123006914398727

A433SER -0.121433934905817

A434PHE 1.80030353383856

A435LEU 0.649342049304658

A436GLN -0.89758636253417

A437LEU 0.265897519939166

A438THR 0.381550517674361

A439GLU -0.152649771764529

A440TYR 0.930477006805302

A441SER -0.644363921783986

A442ARG 1.00206429186638

A443GLU -0.6645847465341

A444GLU -0.61325061759861

A445ILE 0.754880925553586

A446LEU 0.0934469958458323

A447GLY -0.934918517481744

A448ARG 0.148071406801129

A449ASN 0.617371737168174

A450CYS 0.397101990916433

A451ARG -0.0152966762619156

A452PHE 0.565682345624904

A453LEU 0.829756806792474

A454GLN 0.733046310757908

A455GLY -0.875998602993334

A456PRO -1.33872697210984

A457GLU -0.904570420997888

A458THR 0.429632231598054

A459ASP -0.480591029167991

A460ARG -0.278957898432306

A461ALA -1.00313866451242

A462THR 0.0603068930775076

A463VAL 0.516207588309985

A464ARG -0.568682085992583

A465LYS -0.139163359508627

A466ILE 1.21067470120738

A467ARG 0.244226660762771

A468ASP -0.254992173479866

A469ALA 0.0373256783365721

A470ILE 0.602670637596773

A471ASP -0.66796148809619

A472ASN -0.930061660273397

A473GLN -0.300515909868531

A474THR -0.156447189341799

A475GLU -0.425552377315084

A476VAL 0.247705347919263

A477THR -0.260235452947429

A478VAL 0.209078452345949

A479GLN 0.33899674540441

A480LEU 1.11285936061599

A481ILE 0.884592474781438

A482ASN 1.20015540189360

A483TYR 1.38853971069123

A484THR 0.290898028817739

A485LYS -0.657587126030104

A486SER -1.34201913737311

A487GLY -1.36772270225305

A488LYS -0.660618089676994

A489LYS -0.407300937441687

A490PHE 1.41030545110003

A491TRP 1.03682187882449

A492ASN 1.32860302145731

A493LEU 1.00341983546708

A494PHE 1.94565028735919

A495HIS 1.63981807330059

A496LEU 1.40884956249827

A497GLN 0.906441633580395

A498PRO 0.456553549698956

A499MET 0.653149496433857

A500ARG -0.120526048880431

A501ASP -0.611990211162291

A502GLN -1.70068886776974

A503LYS -1.60407822746584

A504GLY -1.46476645626434

A505ASP -0.92712074422402

A506VAL -0.0491255729173749

A507GLN 0.0259512366355419

A508TYR 1.08437739923462

A509PHE 1.73178634894606

A510ILE 1.44430616621727

A511GLY 0.25647324619361

A512VAL 1.18644389700412

A513GLN 1.30515258109174

A514LEU 0.113273269776542

A515ASP -0.492522165619187

A516GLY -1.00010842992753

A517THR -1.42272442923268

A518GLU -1.0782799828096

A519HIS -0.763693512645911

A520VAL -0.310668947044513

A521ARG -1.22103582123807

A522ASP -1.31026627774342

A523ALA -1.28292503644379

A524ALA -0.951749209877487

A525GLU -0.121520735399087

A526ARG -0.824242469129899

A527GLU -0.843020232323048

A528GLY -0.636842511932689

A529VAL -0.0606389642166723

A530MET -0.782695667768366

A531LEU -0.405545875723141

A532ILE 1.0279071221251

A533LYS -0.0111193307937195

A534LYS -0.582885532736318

A535THR 0.32104745369303

A536ALA 0.307898278509252

A537GLU -0.302927213730621

A538ASN -0.381413298074692

A539ILE 0.787213167186317

A540ASP 0.115520494741043

A541GLU -0.897619692666337

A542ALA -0.764813795001322

A543ALA -0.358160984860673

A544LYS -0.581033815632993

A545GLU -1.63325202993896

A546LEU -0.932671330486708

**[GPCR:2j4y, mean 517.24, sd 171.56]**

A108THR -1.19602604219291

A109GLY -0.41380504263382

A10TYR 1.52761795416320

A110CYS 0.752078204519417

A111ASN 0.375062881880481

A112LEU 0.443158882208746

A113GLU 2.10161792765817

A114GLY 0.344722522338351

A115PHE 1.49289431285665

A116PHE 1.31946317138381

A117ALA 0.462725007604451

A118THR 1.30184169622694

A119LEU 1.34748601252026

A11VAL 0.997620656742586

A120GLY 0.0959302735460578

A121GLY -0.0564866657775626

A122GLU 1.86906196154358

A123ILE 1.42283485705818

A124ALA 0.129188985079437

A125LEU 0.9425165656328

A126TRP 2.22883503944155

A127SER 0.446282090651705

A128LEU 0.72701117012295

A129VAL 0.376710287247656

A12PRO 0.596793067236861

A130VAL 0.259734108274348

A131LEU 0.479837824600152

A132ALA 0.01290525981533

A133ILE -0.281298989254462

A134GLU 0.215257640853344

A135ARG 1.06909890301284

A136TYR 0.644942364311822

A137VAL -0.409091569078857

A138VAL -0.513330188253276

A139VAL 0.438806020462564

A13PHE 0.542599480324869

A140CYS -0.143343776228551

A141LYS -0.827887432077255

A142PRO -1.44794121133871

A143MET -2.01751323164991

A144SER -1.91398110546279

A145ASN -1.88147840312218

A146PHE -1.76660075740638

A147ARG -1.77931781956854

A148PHE -0.454024682533341

A149GLY -1.43933056792535

A14SER -0.303738067002066

A150GLU -1.08358276826469

A151ASN -1.25022376443667

A152HIS -0.75390906728214

A153ALA -0.469090247636684

A154ILE -0.679434835945024

A155MET -1.08104473864929

A156GLY -0.836750616153982

A157VAL 0.00688823614638859

A158ALA -1.03960853237377

A159PHE 0.201253713422346

A15ASN -0.377228128793915

A160THR 1.01596022250764

A161TRP 0.848343038218395

A162VAL -0.740672682797078

A163MET -0.00365043214982636

A164ALA 0.550529317826753

A165LEU 0.0401579628476051

A166ALA -0.296214978236103

A167CYS 0.839606930303874

A168ALA 0.532918705372523

A169ALA -0.351785381991248

A16LYS -1.53208412998667

A170PRO 0.333923641749528

A171PRO 0.968038532486058

A172LEU -0.232411599235085

A173VAL -1.32208625258127

A174GLY -1.05237877410450

A175TRP 1.10410664264673

A176SER 0.888905348214965

A177ARG 1.31681698276771

A178TYR 2.71356059853267

A179ILE 1.32229535012799

A17THR -1.46621789205191

A180PRO 1.23275172246452

A181GLU 1.76780624156762

A182GLY 0.262063192543007

A183MET 1.13628802349129

A184GLN 1.64376357494708

A185CYS 0.938415287114376

A186SER 1.06703325588951

A187CYS 1.08984353490941

A188GLY 0.256163668652968

A189ILE 1.38579522503244

A18GLY -1.63219098980412

A190ASP 1.17399167257488

A191TYR 1.82162877885347

A192TYR 1.93858717949050

A193THR 0.0981525885951652

A194PRO -0.735113400665371

A195HIS -0.0851355276865353

A196GLU -1.06913155193904

A197GLU -1.33172756356216

A198THR 0.0663395759062465

A199ASN -0.364209953302295

A19VAL -0.68732676695547

A1AME -1.41767022884670

A200ASN 1.43842156153372

A201GLU -0.171965101880518

A202SER -0.531411927933711

A203PHE 2.10414554354164

A204VAL 0.665478560578701

A205ILE -0.462811321493777

A206TYR 0.949915254052763

A207MET 1.05276552984993

A208PHE 0.88116653106837

A209VAL -0.508100278990892

A20VAL 0.279454725991311

A210VAL -0.115414321062452

A211HIS 1.53324899330080

A212PHE 0.544055614783598

A213ILE -0.443904742087837

A214ILE -0.280924764418421

A215PRO 0.494464893511869

A216LEU -0.0310982715812945

A217ILE -0.807523538874154

A218VAL -0.223470188689414

A219ILE 0.518411038336953

A21ARG -0.133281359865940

A220PHE -0.937377247011505

A221PHE -0.834873046304601

A222CYS 0.0164066293057652

A223TYR -0.206497720793922

A224GLY -1.32764810408952

A225GLN -0.188996828174232

A226LEU 0.522970123722524

A227VAL -0.94985583640972

A228PHE -0.500251634276602

A229THR 0.0309484022110093

A22SER -0.0444695664161992

A230VAL -0.744805667542214

A231LYS -1.38360767688856

A232GLU -1.14252865120637

A233ALA -0.673310688647814

A234ALA -0.785734174652909

A235ALA -1.41402767833621

A236GLN -1.47580328753449

A237GLN -0.738818390352099

A238GLN -1.76001894126534

A239GLU -0.414771307051915

A23PRO 1.13494881374214

A240SER -1.76321259325072

A241ALA -1.84585098728042

A242THR -1.7964433232218

A243THR -0.994055260967101

A244GLN -0.335377365684149

A245LYS -1.13405700426238

A246ALA -1.23513483851859

A247GLU -0.354806873028714

A248LYS -0.443903407362315

A249GLU -0.328453819122640

A24PHE 1.10569890057036

A250VAL -0.0849650501836055

A251THR 0.00743241219357848

A252ARG -0.723316171797015

A253MET 0.465495029318179

A254VAL 0.346118903017319

A255ILE -0.264579468973722

A256ILE -0.486659223312244

A257MET 0.458865191237392

A258VAL -0.038697924193096

A259ILE -0.622621302184997

A25GLU -0.746903160717502

A260ALA -0.546801749662958

A261PHE 1.16098521227737

A262LEU -0.474448690005792

A263ILE -0.791075222205679

A264CYS 0.304314734143153

A265TRP 2.20897194106373

A266LEU -0.240546316170905

A267PRO -0.0927832883156162

A268TYR 2.29327666215459

A269ALA -0.284823621193914

A26ALA -0.660164090508495

A270GLY -1.02753911860780

A271VAL 0.428408817309091

A272ALA 0.216825630790258

A273PHE -0.0727324746853251

A274TYR 0.158547959889346

A275ILE 0.921895205484572

A276PHE 0.759703674704252

A277THR -0.997193385644512

A278HIS -0.848163445356728

A279GLN -0.342702654749321

A27PRO -0.247264782948987

A280GLY -1.65602410245956

A281SER -1.10946686791912

A282CYS -1.29916353068625

A283PHE 0.698557002255315

A284GLY -0.947610354968214

A285PRO 0.187071292474235

A286ILE -0.533473464859156

A287PHE 0.395925908947157

A288MET 1.50726090488878

A289THR 0.747800546579106

A28GLN 1.47258383675372

A290ILE -0.333191486747927

A291PRO 0.700666414407754

A292ALA 0.249951194111848

A293PHE 1.38501112518047

A294PHE 0.239407376719252

A295ALA 0.139939066666249

A296LYR 6.0974496200904

A297THR -0.0761936894045686

A298SER 0.498379449173728

A299ALA 0.407084372945752

A29TYR 0.213461226482768

A2CYS -0.662757403234831

A300VAL 0.329548425354366

A301TYR 0.718866296702336

A302ASN 0.552665576844329

A303PRO 0.725474155668825

A304VAL -0.546391453156335

A305ILE 0.215430047810192

A306TYR 1.3877622759778

A307ILE 0.106149465909672

A308MET -1.25415134260483

A309MET -0.371768790177423

A30TYR 0.677629769351774

A310ASN 0.183239194176116

A311LYS -1.14161086842155

A312GLN -0.677359284403706

A313PHE 1.31638527995207

A314ARG -0.0339236030776501

A315ASN -0.692463644327313

A316CYS -0.299063559016338

A317MET 0.00868962926929868

A318VAL -0.3949492735538

A319THR -0.134810368791204

A31LEU 0.472141145898965

A320THR -0.294908084502368

A321LEU -1.15367203769294

A322CYS -1.23834262395639

A323CYS -1.63001478749444

A324GLY -1.67527660834628

A325LYS -1.58884773992956

A326ASN -1.60723257990429

A327PRO -2.47471981317382

A32ALA -0.692636530477124

A33GLU -1.03525371905207

A34PRO -0.902995024578006

A35TRP -1.01663667052602

A36GLN -0.259245723669855

A37PHE 1.77632417109171

A38SER -0.559516981021711

A39MET -0.760357250692361

A3GLY -0.650379537070146

A40LEU 0.616622236499424

A41ALA -0.108240084155785

A42ALA -0.756385550512708

A43TYR 0.304393857853806

A44MET 1.14983013123485

A45PHE -0.292456320548288

A46LEU -0.575648841948234

A47LEU 0.497489760659209

A48ILE 0.559582400009241

A49MET -0.882651436358596

A4THR 0.136239627336703

A50LEU -0.511317974762112

A51GLY -0.212541995325926

A52PHE -0.141330457420306

A53PRO -0.583629215443269

A54ILE 0.129881307642467

A55ASN 1.31968821576823

A56PHE -0.225689631110815

A57LEU 0.0330509339210837

A58THR 0.669634497527625

A59LEU 0.139159177335499

A5GLU -0.62489218325523

A60TYR -0.346731591125424

A61VAL 0.481364224469325

A62THR 0.224593053982261

A63VAL -1.05143739809761

A64GLN -0.972607817281434

A65HIS 0.0312950375535365

A66LYS -1.50349393391553

A67LYS -1.37546770624955

A68LEU 0.0421691354731473

A69ARG -0.34137916715099

A6GLY -1.42350555949041

A70THR -0.91103628907857

A71PRO -0.256254528072739

A72LEU -0.0271588880364266

A73ASN 0.276320237510632

A74TYR 0.650891552516496

A75ILE 0.738800625480087

A76LEU 0.689433396271927

A77LEU 0.309515705473194

A78ASN 0.587669594562109

A79LEU 1.12866946636352

A7PRO -1.50645697988906

A80ALA 0.155474317672102

A81VAL -0.102648112779965

A82ALA 0.417689977798191

A83ASP 1.35369145711955

A84LEU 0.836679244514206

A85PHE 0.261417984838866

A86MET 1.36216550498988

A87VAL 0.704216374421896

A88PHE -0.293193988509544

A89GLY -0.439972388724625

A8ASN -0.347780798087231

A90GLY 0.0478724797401095

A91PHE 1.81853309878311

A92THR -0.221385134589619

A93THR 0.539168972032714

A94THR 1.04612713795939

A95LEU 0.748389237927836

A96TYR 0.0586141153032369

A97THR 0.916696542794962

A98SER 0.740022862941499

A99LEU -0.237776519302886

A9PHE 1.28964219261766
