# Supplementary material for: Modeling allosteric signal propagation using protein structure networks
Source: BMC Bioinformatics. 2011 Feb 15;12(Suppl 1):S23. doi: 10.1186/1471-2105-12-S1-S23 (PMC3044278; doi:10.1186/1471-2105-12-S1-S23)
Supplement: Additional file 3 — Thirteen linear regression models of the average EVT and ΔΔG values for each protein structure network [file 1471-2105-12-S1-S23-S3.doc]

Supp3. Linear regression models between averaged EVT and ∆∆G value in the weighted network.

| PDB | Distance cut-off | Correlation coefficient | Residual standard error | p-val |
| --- | --- | --- | --- | --- |
| 1cbw | 3.0 | 0.891 | 0.383 | 0.0172 |
|  | 3.5 | 0.834 | 0.466 | 0.0393 |
|  | 4.0 | 0.968 | 0.210 | 0.0015 |
|  | 4.5 | 0.925 | 0.321 | 0.0083 |
|  | 5.0 | 0.951 | 0.260 | 0.0035 |
|  | 5.5 | 0.917 | 0.337 | 0.0101 |
|  | 6.0 | 0.921 | 0.328 | 0.0091 |
|  | 6.5 | 0.938 | 0.292 | 0.0056 |
|  | 7.0 | 0.915 | 0.339 | 0.0104 |
|  | 7.5 | 0.903 | 0.362 | 0.0136 |
|  | 8.0 | 0.893 | 0.380 | 0.0167 |
|  | 8.5 | 0.900 | 0.368 | 0.0145 |
|  | 9.0 | 0.913 | 0.343 | 0.0109 |
|  | 9.5 | 0.911 | 0.347 | 0.0114 |
|  | 10.0 | 0.907 | 0.356 | 0.0127 |
|  | 10.5 | 0.905 | 0.358 | 0.0131 |
|  | 11.0 | 0.896 | 0.375 | 0.0158 |
|  | 11.5 | 0.882 | 0.397 | 0.0200 |
|  | 12.0 | 0.880 | 0.400 | 0.0207 |
| 1brs | 3.0 | 0.108 | 2.460 | 0.7518 |
|  | 3.5 | 0.291 | 2.368 | 0.3851 |
|  | 4.0 | 0.838 | 1.351 | 0.0013 |
|  | 4.5 | 0.760 | 1.610 | 0.0067 |
|  | 5.0 | 0.756 | 1.620 | 0.0071 |
|  | 5.5 | 0.732 | 1.685 | 0.0104 |
|  | 6.0 | 0.755 | 1.623 | 0.0072 |
|  | 6.5 | 0.693 | 1.784 | 0.0181 |
|  | 7.0 | 0.694 | 1.782 | 0.0178 |
|  | 7.5 | 0.688 | 1.796 | 0.0192 |
|  | 8.0 | 0.694 | 1.782 | 0.0178 |
|  | 8.5 | 0.698 | 1.771 | 0.0168 |
|  | 9.0 | 0.700 | 1.767 | 0.0164 |
|  | 9.5 | 0.706 | 1.754 | 0.0153 |
|  | 10.0 | 0.706 | 1.753 | 0.0152 |
|  | 10.5 | 0.686 | 1.801 | 0.0198 |
|  | 11.0 | 0.697 | 1.775 | 0.0172 |
|  | 11.5 | 0.693 | 1.783 | 0.0180 |
|  | 12.0 | 0.688 | 1.796 | 0.0193 |
| 1fcc | 3.0 | 0.314 | 1.859 | 0.4485 |
|  | 3.5 | 0.527 | 1.665 | 0.1798 |
|  | 4.0 | 0.692 | 1.414 | 0.0572 |
|  | 4.5 | 0.850 | 1.030 | 0.0075 |
|  | 5.0 | 0.870 | 0.930 | 0.0039 |
|  | 5.5 | 0.867 | 0.976 | 0.0053 |
|  | 6.0 | 0.836 | 1.075 | 0.0097 |
|  | 6.5 | 0.809 | 1.152 | 0.0151 |
|  | 7.0 | 0.814 | 1.137 | 0.0139 |
|  | 7.5 | 0.847 | 1.040 | 0.0079 |
|  | 8.0 | 0.840 | 1.062 | 0.0090 |
|  | 8.5 | 0.825 | 1.107 | 0.0117 |
|  | 9.0 | 0.833 | 1.082 | 0.0102 |
|  | 9.5 | 0.832 | 1.087 | 0.0105 |
|  | 10.0 | 0.813 | 1.140 | 0.0141 |
|  | 10.5 | 0.803 | 1.167 | 0.0164 |
|  | 11.0 | 0.794 | 1.191 | 0.0187 |
|  | 11.5 | 0.782 | 1.221 | 0.0219 |
|  | 12.0 | 0.776 | 1.235 | 0.0236 |
| 1f47 | 3.0 | 0.462 | 0.910 | 0.2496 |
|  | 3.5 | 0.322 | 0.972 | 0.4372 |
|  | 4.0 | 0.620 | 0.805 | 0.1010 |
|  | 4.5 | 0.578 | 0.837 | 0.1331 |
|  | 5.0 | 0.654 | 0.776 | 0.0785 |
|  | 5.5 | 0.623 | 0.803 | 0.0990 |
|  | 6.0 | 0.590 | 0.829 | 0.1239 |
|  | 6.5 | 0.568 | 0.845 | 0.1422 |
|  | 7.0 | 0.566 | 0.846 | 0.1433 |
|  | 7.5 | 0.575 | 0.840 | 0.1359 |
|  | 8.0 | 0.533 | 0.868 | 0.1737 |
|  | 8.5 | 0.517 | 0.879 | 0.1898 |
|  | 9.0 | 0.502 | 0.888 | 0.2048 |
|  | 9.5 | 0.518 | 0.878 | 0.1880 |
|  | 10.0 | 0.538 | 0.865 | 0.1691 |
|  | 10.5 | 0.536 | 0.866 | 0.1705 |
|  | 11.0 | 0.539 | 0.864 | 0.1676 |
|  | 11.5 | 0.527 | 0.872 | 0.1795 |
|  | 12.0 | 0.515 | 0.880 | 0.1919 |
| 1dan | 3.0 | 0.030 | 0.850 | 0.9138 |
|  | 3.5 | 0.047 | 0.850 | 0.8619 |
|  | 4.0 | 0.455 | 0.757 | 0.0763 |
|  | 4.5 | 0.500 | 0.737 | 0.0486 |
|  | 5.0 | 0.486 | 0.743 | 0.0560 |
|  | 5.5 | 0.508 | 0.733 | 0.0445 |
|  | 6.0 | 0.511 | 0.731 | 0.0431 |
|  | 6.5 | 0.471 | 0.750 | 0.0655 |
|  | 7.0 | 0.501 | 0.736 | 0.0478 |
|  | 7.5 | 0.528 | 0.722 | 0.0357 |
|  | 8.0 | 0.550 | 0.710 | 0.0274 |
|  | 8.5 | 0.560 | 0.705 | 0.0241 |
|  | 9.0 | 0.565 | 0.701 | 0.0225 |
|  | 9.5 | 0.574 | 0.696 | 0.0200 |
|  | 10.0 | 0.564 | 0.703 | 0.0230 |
|  | 10.5 | 0.564 | 0.702 | 0.0229 |
|  | 11.0 | 0.560 | 0.705 | 0.0241 |
|  | 11.5 | 0.558 | 0.705 | 0.0245 |
|  | 12.0 | 0.555 | 0.707 | 0.0255 |
| 3hfm | 3.0 | 0.246 | 2.556 | 0.2584 |
|  | 3.5 | 0.054 | 2.632 | 0.8064 |
|  | 4.0 | 0.279 | 2.531 | 0.1980 |
|  | 4.5 | 0.503 | 2.278 | 0.0145 |
|  | 5.0 | 0.573 | 2.159 | 0.0042 |
|  | 5.5 | 0.638 | 2.030 | 0.0011 |
|  | 6.0 | 0.689 | 1.910 | 0.0003 |
|  | 6.5 | 0.691 | 1.905 | 0.0003 |
|  | 7.0 | 0.676 | 1.943 | 0.0004 |
|  | 7.5 | 0.691 | 1.905 | 0.0003 |
|  | 8.0 | 0.664 | 1.970 | 0.0005 |
|  | 8.5 | 0.654 | 1.993 | 0.0007 |
|  | 9.0 | 0.668 | 1.961 | 0.0005 |
|  | 9.5 | 0.668 | 1.962 | 0.0005 |
|  | 10.0 | 0.671 | 1.954 | 0.0005 |
|  | 10.5 | 0.666 | 1.965 | 0.0005 |
|  | 11.0 | 0.668 | 1.961 | 0.0005 |
|  | 11.5 | 0.664 | 1.971 | 0.0006 |
|  | 12.0 | 0.662 | 1.976 | 0.0006 |
| 1ahw | 3.0 | 0.695 | 1.203 | 0.0832 |
|  | 3.5 | 0.240 | 1.624 | 0.6050 |
|  | 4.0 | 0.227 | 1.630 | 0.6250 |
|  | 4.5 | 0.204 | 1.638 | 0.6613 |
|  | 5.0 | 0.431 | 1.510 | 0.3347 |
|  | 5.5 | 0.523 | 1.426 | 0.2282 |
|  | 6.0 | 0.603 | 1.335 | 0.1519 |
|  | 6.5 | 0.656 | 1.263 | 0.1099 |
|  | 7.0 | 0.670 | 1.242 | 0.0997 |
|  | 7.5 | 0.700 | 1.195 | 0.0801 |
|  | 8.0 | 0.728 | 1.147 | 0.0635 |
|  | 8.5 | 0.723 | 1.156 | 0.0665 |
|  | 9.0 | 0.740 | 1.125 | 0.0572 |
|  | 9.5 | 0.745 | 1.116 | 0.0546 |
|  | 10.0 | 0.746 | 1.114 | 0.0541 |
|  | 10.5 | 0.740 | 1.126 | 0.0574 |
|  | 11.0 | 0.732 | 1.139 | 0.0612 |
|  | 11.5 | 0.728 | 1.147 | 0.0636 |
|  | 12.0 | 0.705 | 1.187 | 0.0770 |
| 1a22 | 3.0 | 0.234 | 1.093 | 0.0913 |
|  | 3.5 | 0.099 | 1.119 | 0.4831 |
|  | 4.0 | 0.224 | 1.096 | 0.1075 |
|  | 4.5 | 0.325 | 1.063 | 0.0174 |
|  | 5.0 | 0.380 | 1.040 | 0.0050 |
|  | 5.5 | 0.422 | 1.020 | 0.0016 |
|  | 6.0 | 0.449 | 1.005 | 0.0007 |
|  | 6.5 | 0.486 | 0.983 | 0.0002 |
|  | 7.0 | 0.478 | 0.988 | 0.0003 |
|  | 7.5 | 0.491 | 0.980 | 0.0002 |
|  | 8.0 | 0.521 | 0.960 | 6.443e-05 |
|  | 8.5 | 0.526 | 0.956 | 5.206e-05 |
|  | 9.0 | 0.538 | 0.948 | 3.256e-05 |
|  | 9.5 | 0.542 | 0.945 | 2.765e-05 |
|  | 10.0 | 0.549 | 0.940 | 2.041e-05 |
|  | 10.5 | 0.558 | 0.934 | 1.441e-05 |
|  | 11.0 | 0.554 | 0.937 | 1.712e-05 |
|  | 11.5 | 0.551 | 0.939 | 1.901e-05 |
|  | 12.0 | 0.550 | 0.939 | 1.973e-05 |
| 1bxi | 3.0 | 0.199 | 1.622 | 0.4439 |
|  | 3.5 | 0.117 | 1.644 | 0.6560 |
|  | 4.0 | 0.187 | 1.626 | 0.4714 |
|  | 4.5 | 0.394 | 1.521 | 0.1174 |
|  | 5.0 | 0.469 | 1.461 | 0.0574 |
|  | 5.5 | 0.513 | 1.420 | 0.0351 |
|  | 6.0 | 0.580 | 1.348 | 0.0146 |
|  | 6.5 | 0.603 | 1.321 | 0.0105 |
|  | 7.0 | 0.610 | 1.311 | 0.0093 |
|  | 7.5 | 0.620 | 1.299 | 0.0080 |
|  | 8.0 | 0.618 | 1.301 | 0.0082 |
|  | 8.5 | 0.653 | 1.253 | 0.0045 |
|  | 9.0 | 0.653 | 1.253 | 0.0045 |
|  | 9.5 | 0.656 | 1.249 | 0.0043 |
|  | 10.0 | 0.655 | 1.250 | 0.0043 |
|  | 10.5 | 0.644 | 1.266 | 0.0052 |
|  | 11.0 | 0.628 | 1.288 | 0.0069 |
|  | 11.5 | 0.629 | 1.287 | 0.0069 |
|  | 12.0 | 0.632 | 1.283 | 0.0065 |
| 1jrh | 3.0 | 0.180 | 1.352 | 0.3689 |
|  | 3.5 | 0.225 | 1.339 | 0.2600 |
|  | 4.0 | 0.175 | 1.353 | 0.3823 |
|  | 4.5 | 0.364 | 1.28 | 0.0620 |
|  | 5.0 | 0.392 | 1.264 | 0.0433 |
|  | 5.5 | 0.397 | 1.261 | 0.0402 |
|  | 6.0 | 0.417 | 1.249 | 0.0303 |
|  | 6.5 | 0.402 | 1.258 | 0.0378 |
|  | 7.0 | 0.393 | 1.263 | 0.0423 |
|  | 7.5 | 0.380 | 1.271 | 0.0508 |
|  | 8.0 | 0.340 | 1.292 | 0.0828 |
|  | 8.5 | 0.328 | 1.298 | 0.0947 |
|  | 9.0 | 0.308 | 1.307 | 0.1177 |
|  | 9.5 | 0.289 | 1.315 | 0.1435 |
|  | 10.0 | 0.276 | 1.321 | 0.1629 |
|  | 10.5 | 0.258 | 1.328 | 0.1941 |
|  | 11.0 | 0.248 | 1.331 | 0.2124 |
|  | 11.5 | 0.232 | 1.337 | 0.2443 |
|  | 12.0 | 0.220 | 1.340 | 0.2702 |
| 1dvf | 3.0 | 0.216 | 1.267 | 0.3896 |
|  | 3.5 | 0.190 | 1.274 | 0.4509 |
|  | 4.0 | 0.031 | 1.297 | 0.9040 |
|  | 4.5 | 0.187 | 1.274 | 0.4565 |
|  | 5.0 | 0.166 | 1.279 | 0.5105 |
|  | 5.5 | 0.286 | 1.243 | 0.2500 |
|  | 6.0 | 0.320 | 1.229 | 0.1949 |
|  | 6.5 | 0.368 | 1.206 | 0.1328 |
|  | 7.0 | 0.337 | 1.221 | 0.1713 |
|  | 7.5 | 0.357 | 1.212 | 0.1459 |
|  | 8.0 | 0.355 | 1.213 | 0.1479 |
|  | 8.5 | 0.357 | 1.212 | 0.1460 |
|  | 9.0 | 0.364 | 1.208 | 0.1373 |
|  | 9.5 | 0.359 | 1.211 | 0.1429 |
|  | 10.0 | 0.357 | 1.212 | 0.1453 |
|  | 10.5 | 0.333 | 1.223 | 0.1771 |
|  | 11.0 | 0.341 | 1.219 | 0.1655 |
|  | 11.5 | 0.340 | 1.220 | 0.1680 |
|  | 12.0 | 0.341 | 1.220 | 0.1663 |
| 1gc1 | 3.0 | 0.082 | 0.516 | 0.7537 |
|  | 3.5 | 0.100 | 0.515 | 0.7000 |
|  | 4.0 | 0.080 | 0.516 | 0.7605 |
|  | 4.5 | 0.273 | 0.498 | 0.2887 |
|  | 5.0 | 0.176 | 0.510 | 0.4989 |
|  | 5.5 | 0.259 | 0.500 | 0.3156 |
|  | 6.0 | 0.248 | 0.502 | 0.3376 |
|  | 6.5 | 0.316 | 0.492 | 0.2171 |
|  | 7.0 | 0.309 | 0.493 | 0.2268 |
|  | 7.5 | 0.319 | 0.491 | 0.2124 |
|  | 8.0 | 0.309 | 0.493 | 0.2281 |
|  | 8.5 | 0.274 | 0.498 | 0.2868 |
|  | 9.0 | 0.237 | 0.503 | 0.3597 |
|  | 9.5 | 0.197 | 0.508 | 0.4480 |
|  | 10.0 | 0.195 | 0.508 | 0.4527 |
|  | 10.5 | 0.160 | 0.511 | 0.5401 |
|  | 11.0 | 0.141 | 0.513 | 0.5895 |
|  | 11.5 | 0.129 | 0.514 | 0.6205 |
|  | 12.0 | 0.121 | 0.514 | 0.6436 |
| 1vfb | 3.0 | 0.341 | 1.204 | 0.2540 |
|  | 3.5 | 0.133 | 1.269 | 0.6650 |
|  | 4.0 | 0.058 | 1.278 | 0.8509 |
|  | 4.5 | 0.123 | 1.271 | 0.6882 |
|  | 5.0 | 0.132 | 1.269 | 0.6667 |
|  | 5.5 | 0.138 | 1.268 | 0.6518 |
|  | 6.0 | 0.235 | 1.245 | 0.4406 |
|  | 6.5 | 0.281 | 1.229 | 0.3532 |
|  | 7.0 | 0.319 | 1.214 | 0.2885 |
|  | 7.5 | 0.394 | 1.177 | 0.1831 |
|  | 8.0 | 0.459 | 1.137 | 0.1144 |
|  | 8.5 | 0.495 | 1.113 | 0.0858 |
|  | 9.0 | 0.524 | 1.091 | 0.0663 |
|  | 9.5 | 0.563 | 1.058 | 0.0451 |
|  | 10.0 | 0.576 | 1.047 | 0.0394 |
|  | 10.5 | 0.581 | 1.042 | 0.0372 |
|  | 11.0 | 0.594 | 1.030 | 0.0324 |
|  | 11.5 | 0.593 | 1.031 | 0.0328 |
|  | 12.0 | 0.593 | 1.031 | 0.0328 |
